# The colonoscopic vacuum model–simulating biomechanical restrictions to provide a realistic colonoscopy training environment

**DOI:** 10.1007/s11548-022-02792-z

**Published:** 2022-11-23

**Authors:** Jana Steger, Christina Kwade, Maximilian Berlet, Roman Krumpholz, Stefanie Ficht, Dirk Wilhelm, Petra Mela

**Affiliations:** 1 Research Group Minimally-Invasive Interdisciplinary Therapeutical Intervention (MITI), Klinikum Rechts Der Isar, Technical University of Munich, Munich, Germany; 2 Chair of Medical Materials and Implants, Department of Mechanical Engineering and Munich Institute of Biomedical Engineering, TUM School of Engineering and Design, Technical University of Munich, Munich, Germany; 3 Clinic and Policlinic for Surgery, Faculty of medicine, Technical University of Munich, Munich, Germany

**Keywords:** Colonoscopy, Ex vivo, Simulator, Additive manufacturing

## Abstract

**Introduction:**

Practicing endoscopic procedures is fundamental for the education of clinicians and the benefit of patients. Despite a diverse variety of model types, there is no system simulating anatomical restrictions and variations in a flexible and atraumatic way. Our goal was to develop and validate a new modelling approach for adhesion forces between colon and abdominal wall.

**Methods:**

An inlay for a standard mechanical trainer was designed and 3D printed. Colon specimens were fixed to the inlay along colon ascendens (CA) and colon descendens (CD) by a vacuum. Our system, which we refer to as Colonoscopy Vacuum Model (CoVaMo), was validated with 11 test persons with varying level of expertise. Each performed one colonoscopy and one polypectomy in the CoVaMo and in the Endoscopic Laparoscopic Interdisciplinary Training Entity (ELITE). Achieved adhesion forces, times required to fulfill different tasks endoscopically and a questionnaire, assessing proximity to reality, were recorded.

**Results:**

Mean adhesion forces of 37 ± 7 N at the CA and 30 ± 15 N at the CD were achieved. Test subjects considered CoVaMo more realistic than ELITE concerning endoscope handling and the overall anatomy. Participants needed statistically significantly more time to maneuver from anus to flexura sinistra in CoVaMo (377 s ± 244 s) than in ELITE (58 s ± 49 s).

**Conclusion:**

We developed a training environment enabling anatomically and procedural realistic colonoscopy training requiring participants to handle all endoscope features in parallel. Fixation forces compare to forces needed to tear pig colon off the mesentery. Workflow and inlay can be adapted to any arbitrary ex vivo simulator.

## Introduction

The invention of flexible endoscopes, which enable atraumatic entry of hollow organs and endoluminal examination, represented a revolutionary step in diagnostic and therapeutic medicine [1]. Endoscopic procedures require trained operators. Historically, endoscopic training was mainly based on mentored supervision during procedures performed on patients, which, especially for novices, may arise ethical concerns [2, 3]. Learning and practicing endoscopic procedures in an environment that is close to reality, offers many advantages for the education of clinicians and allows to fill this gap. Procedure simulations in a risk-free and controlled artificial environment, not only offer the possibility to practice techniques on a frequent basis and to run through different risk scenarios to reduce intraoperative technique-related complications [2, 3], but also support the development and evaluation of new surgical instruments.

## State of the art

Simulators for gastrointestinal endoscopy can be clustered into mechanical, computer-based, live animal and ex vivo models [2, 3]. While mechanical and computer-based simulators are mainly used in early stages of training, live animal and ex vivo models serve for more advanced trainees [2].*Ex Vivo-Models* are simulators consisting of tissue samples or entire organs, obtained from the local slaughterhouse, deposited into a predefined from [2, 4, 5]. They offer several advantages, such as a more realistic device-tissue interaction than mechanical simulators with only artificial materials may provide, cost effectiveness with respect to animal models, and the possibility to validate not only standard procedures (Virtual reality simulation models [6]), but also innovative devices and new techniques. The *Laparoscopic Abdominal Simulator (Limbs & Things Ltd, Sussex Street, St Philips, Bristol BS2 0RA, Vereinigtes Königreich)* is a training model for laparoscopic anastomosis suturing [7, 8]. It comprises an anatomical replica of a human torso, in which animal explants are positioned, and an abdominal wall with incisions for insertion of laparoscopic instruments [7]. Another example is the *Tübinger MIC-Trainer* developed by the *University Hospital Tübingen* and the *Richard Wolf GmbH (Knittlingen, Germany)*, consisting of metallic meshes, three dimensionally shaped according to the dorsal and lateral abdominal walls, a fluid reservoir to catch up organ and cleaning fluids and a neoprene cover to enable trocar insertion. The trainer also comprises an anus reconstruction for the training of stapled anastomoses. [6]

The *EndoExpert Tray (DeLegge Medical, Inc., 4057 Longmarsh, Awendaw, SC 29,429, USA),* the *ColoEASIE-2 and the EASIE-R1 and -R3 (Erlangen Active Simulator for Interventional Endoscopy) (EndoSim, LLC, 41 Main St., Bolton, MA 01,740, USA)* and the Ex Vivo* Colon Model for ESD* are more anatomically alienated models*.* These models are no abdominal replica, but consist of abstracted moldings, that determine the shape of integrated organ specimens^1^ [2, 4, 5]. Gromski et al. used an early prototype of the ColoEASIE-2 in their study for learning colorectal endoscopic submucosal dissection (ESD). This prototype comprises a shaping wire, Which is wrapped around the intestinal specimen at the ascending and descending colon to simulate the immobility in the retroperitoneal areas [5]. Most recently evolved models, such as the commercially available EndoSim models are plastic moldings with transparent covers. While the *EASIE-R1 and –R3* are designed to take-up entire gastrointestinal organ packages, the ColoEASIE-2 is specifically designed for intestinal tissue, shaping a colon geometry.^2^ The EASIE simulators enable the simulation of bleeding [2]. The field of ex vivo-simulators is completed by various models of different research groups. One example is the training box by Brigic et al., which consists of a plastic box with a fabric cover. However, this model represents an even stronger abstraction, as the connection of the colon to the box is the only anchor point, neglecting the interaction with other organs [9]. The Endo *X* Trainer is a portable tray with a molding to position colon tissue. It enables the simulation of bleeding [10]. Retroperitoneal segments of the colon are tacked down by meshes [11].

The field of abdominal trainers is divers, however, most of these models do not aim to provide a mechanically representative depiction of anatomical restrictions [2, 4, 5], such as the fusion with the posterior abdominal wall at colon ascendens (CA) and colon descendens (CD). Due to the strict predefined architecture, there is only little scope for modifications of the anatomy, even though these are highly relevant for achieving a training effect.

The goal of our research was to provide a realistic and flexible training environment for new therapeutic, endoscopic procedures and devices, which will arise in future with respect to the accelerating trend of minimally-invasive surgery. Therefore, we developed an endoscopy simulator, which allows for measurable, modifiable and atraumatic tissue fixation, to realistically simulate varying anatomies and biomechanics, thus adhesion forces to the abdominal wall. A special focus was set on the depiction of procedural complications encountered during colonoscopy, such as loss of orientation within the lumen, hampered advancement due to the colon haustra or loop formation of the flexible endoscope. The use of our model with porcine colon specimens allows to flexibly perform not only diagnostic, but also invasive procedure training, injuring simulator specimen material.

## Materials and methods

### General concept and design

**Fig. 1:**
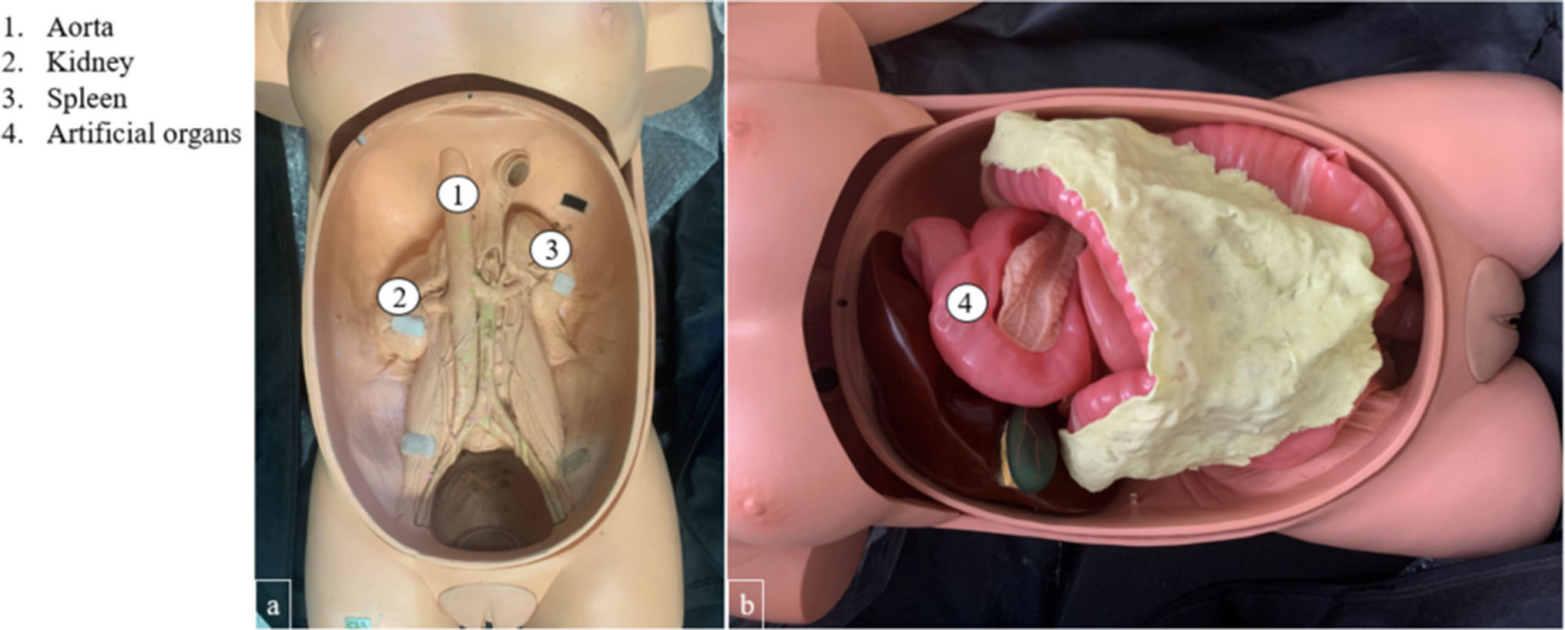
Photographs of the ELITE. **a** Torso; **b** Abdominal, polymeric organs connected to the ELITE

We designed an inlay for the ELITE (CLA, Coburger Lehrmittelanstalt, Coburg, Germany), which is a standard validated mechanical simulator for the training of laparoscopic and endoscopic techniques [12], to introduce a porcine colon and to enable, in this way, a more realistic depiction of colon material properties with respect to instrument gliding behavior, tissue manipulability and bowel wall elasticity. We designed an inlay serving as an anatomy simulating specimen guidance for the ELITE. Porcine tissue is furthermore commonly used for gastrointestinal ex vivo models as it has the highest resemblance of all laboratory animals (except for primates) to human bowel with respect to length, physiology, digestive function and blood flow characteristics [13]. Aorta, spleen, diaphragm, kidneys, vena cava and the iliopsoas musculus are part of the solid torso, which is designed to simulate the three-dimensional appearance of the abdominal cavity made of epoxy resin (Fig. 1a). It furthermore comprises a set of artificial, polymer-based organs (colon, liver, spleen) (Fig. 1b), which can be easily removed from and connected to the body.

**Fig. 2:**
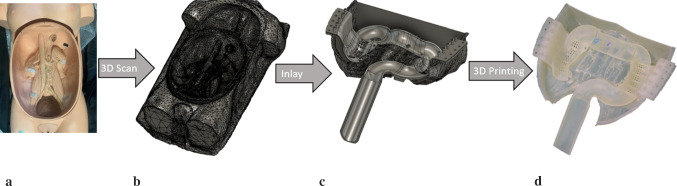
Development and manufacturing process of the CoVaMo inlay. **a** ELITE torso; **b** 3D reconstruction of the ELITE; **c** 3D design of inlay; **d** 3D printed inlay

**Fig. 3:**
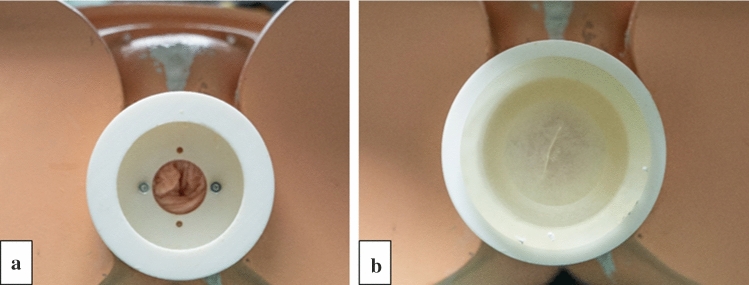
**a** Anus reconstruction port and **b** Silicone component for airtight sealing around the inserted colonoscope (not shown)

The interior of the ELITE was reconstructed by using Autodesk ReCap, Meshmixer (Autodesk, Inc., San Rafael, CA, USA) and Fusion 360 (Autodesk Inc., San Rafael, CA, USA) (Fig. 2), to build up a tight form-fitting inlay for the ELITE corpus (Fig. 3a). The model consists of 12 parts made of Durable resin,^3^ with the SLA printer Formlabs2 (Formlabs GmbH, Berlin, Germany). The parts are assembled and connected with each other by means of puzzle interfaces.

Furthermore, the model comprises a two-part adapter to reconstruct the model’s artificial anus (Fig. 3b). One part is attached to the CoVaMo and protrudes out of the pelvis through a whole between the ELITE’s leg stumps. The intestinal specimen is passed all the way through this hole and the port. The bowel wall is everted and compressed between the adapter halves, as the counterpart is screwed in. The entry point of the endoscope into the model is sealed airtight by a silicone insert (Fig. 3c). In this way, CoVaMo enables the required operation of all endoscope features, including insufflation to establish a stable lumen, aspiration to allow visibility and endoscopic tip position control to maneuver throughout the colon.

### Simulation of anatomical restrictions

In the human organism, the colon is divided into segments. Differentiation is based on the retro- or intraperitoneal location of the colon [14]. These sections are fused with the dorsal abdominal wall. At the sigmoid, left and right flexures, as well as at the transversum, the colon runs intraperitoneally and thus has a greater range of mobility [14].

The model must not only simulate the shape of the colon, but also depict these biomechanical restrictions realistically. This requires a connection between model and tissue, allowing for quick preparation, atraumatic tissue fixation and non-destructive de- and reattachment, also for multiple preparation cycles. Connecting tissue and model via magnets or suction pressure are both options, which meet these requirements. However, magnets already bear some conceptual disadvantages. One of the magnets must be positioned within the colon lumen. As the realizable adhesion forces correlate with the magnet size and as the mutual interaction forces decrease significantly with increasing distance between the components (interposed tissue), magnets of considerable size are required, which are likely to decrease the lumen cross section. Furthermore, they might interfere with endoscopic devices and other magnetic instruments.

The establishment of suction pressure not only avoids both of these drawbacks, but also allows quantification and adaptation of adhesion forces between the colon specimen and inlay. Thus, we simulated the fusion with the abdominal wall, by creating a suction pressure in chambers located underneath the guiding rail. The specimen tissue is placed into the guiding groove, covering holes of different sizes (central holes with 4 mm diameter and holes to both sides with 3 mm diameter). Two suction pumps with a maximum achievable pressure of –80 kPa ($\Delta p$ with respect to atmospheric pressure) are attached to the chambers on both sides of the model (one pump to each side). By variation of the suction pressure, adhesion forces can be tuned. On the side of CA, the total suction surface area is 660 mm^2^ and at CD 640 mm^2^.

**Fig. 4:**
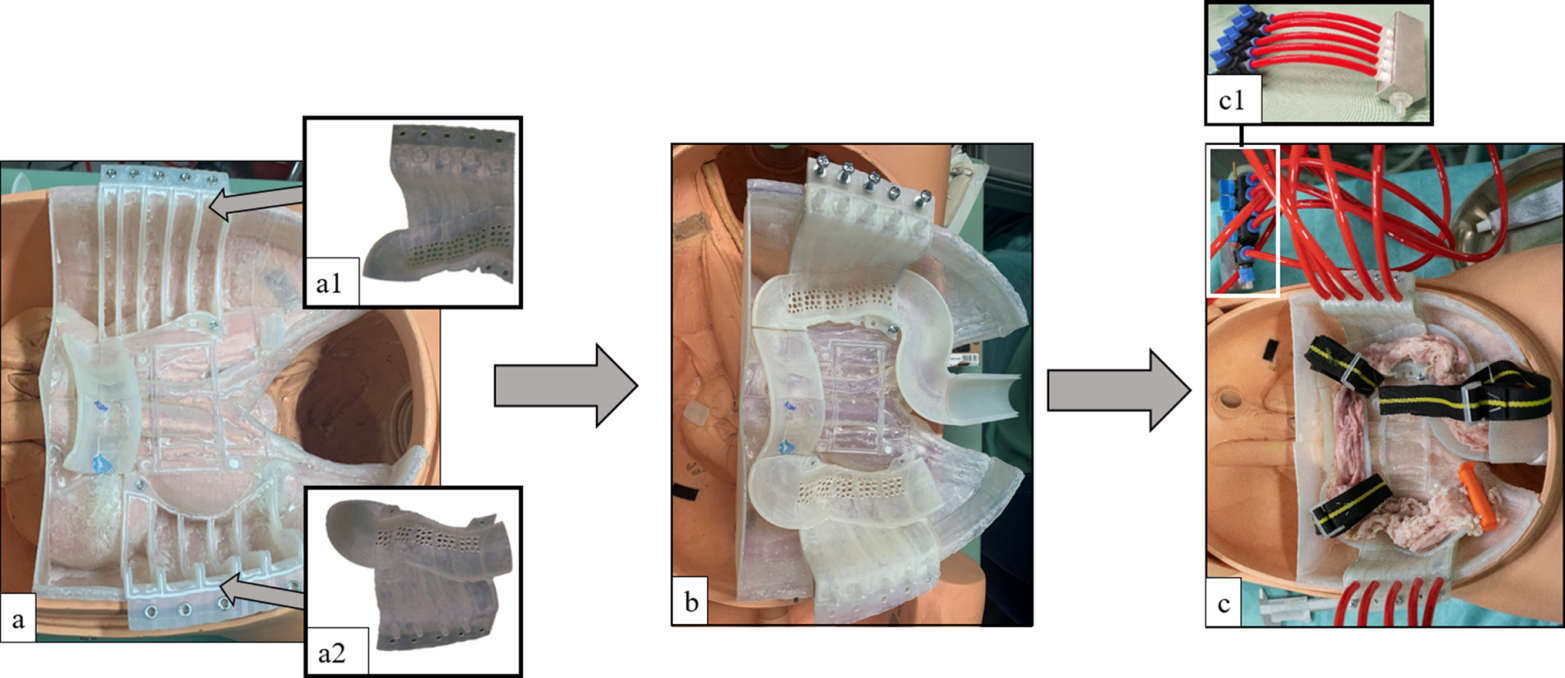
**a** CoVaMo inlay with open chambers at the colon ascendens and colon descendens. **a1** and **a2** Covers of the colon ascendens and colon descendens. **b** CoVaMo with closed cover at the colon ascendens and colon descendens **c** Prepared CoVaMo with colon specimen and straps. **c1** Distributor valve to individually deflate each of the five chambers. Between one, up to five chambers can be activated to simulate anatomical variations of adhesion

**Fig. 5:**
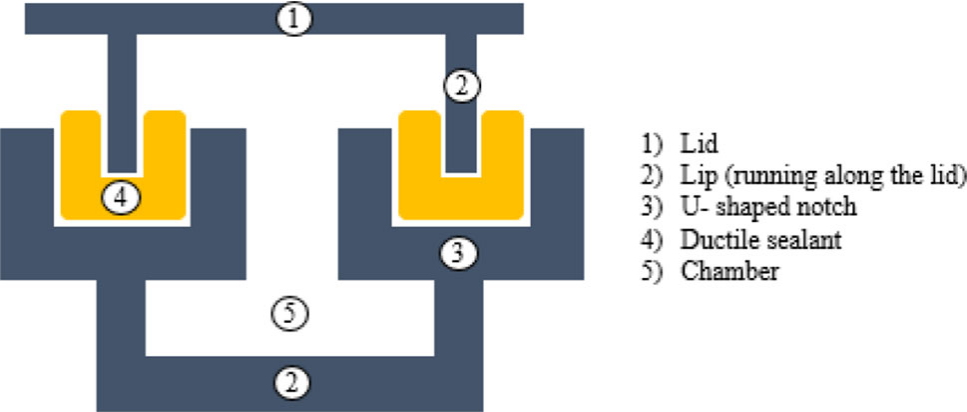
Schematics of lid and body of the inlay to enable airtight closure. The edges of the chambers (5) have an U-shaped notch running along the entire profile (3). The notch contains a ductile sealant (4), into which a lip (2), running along the cover (1) is pressed

To be able to remove fluid and tissue residues, the model consists of a reclosable corpus (form-fit inlay) and a lid (with the positioning groove). Body and lid can be snapped together and opened to facilitate cleaning of the chambers (Fig. 4 a, b, c; Fig. 5).

A U-shaped notch (Fig. 5) runs along the chambers of the inlay base (Fig. 4a), into which the lip, running along the contours of the lid, is inserted. To ensure airtight closure, a highly ductile sealant is positioned in the notch (Fig. 5), and lid and body are compressed with screws (Fig. 4b).

Cavities on both sides are divided into 5 separate chambers (Fig. 4a), each of which can be deflated separately by interposing a distributor valve (Fig. 4c1).

During a standard colonoscopy, an assistant can press against the abdominal wall from the outside to avoid looping of the endoscope during the advancement. Straps (Fig. 4c) at the flexura dextra, flexura sinistra and sigma are used to simulate this additional assistance and counteract the uprighting of the bowel at the intraperitoneal segments.

### Validation of the endoscopic trainer and study design

We validated the model by performing an experimental trial with one medical student, three assistant doctors, five surgeons and two gastrointestinal endoscopy experts of the Klinikum rechts der Isar, at the Technical University of Munich. The new model was assessed in direct comparison with the standard mechanical trainer ELITE. Based on guidelines for the design of validation studies, given by Adler et al., we chose eleven participants to be able to identify at least 84% of potential usability problems and improvement aspects of our training system [15]. The participants were divided into novice, advanced and expert groups, based on the amount of colonoscopies they had performed, according to own specifications. Specifically, *X* corresponding to the amount of performed colonoscopies, defined novices $( {\varvec{X}} < 100)$, advanced operators $(100 \le {\varvec{X}} < 1000)$ and experts $\left( {{\varvec{X}} \ge 1000} \right)$.

**Fig. 6:**
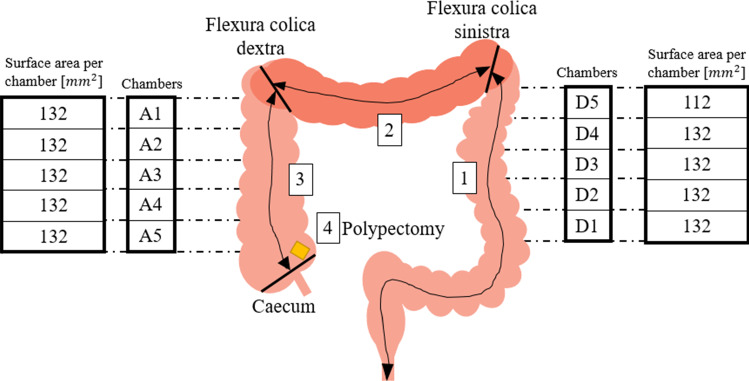
Schematics illustrating the anatomical landmarks, the vacuum chamber segmentation and the suction surface area per chamber. The numbers indicate the tasks for which time was measured for each participant. (1) Advancement from anus to flexura sinsitra; (2) Advancement from flexura sinistra to flexura dextra; (3) Advancement from flexura dextra to caecum; (4) Performing a polypectomy

Several meters of porcine colonic tissue were collected from the local slaughterhouse on each experiment day and stored in a refrigerated bag until usage. The intestine was changed before each test person started their session. Sample sizes of 90 cm length were prepared for each test person. The loop size at the sigma was set to 112 mm, at the flexura sinistra 60.5 mm and 58.5 mm at the flexura dextra (distance from the inlay to the highest point of the stretched loop). The participants used an endoscope from Storz (13801PKS) (Karl Storz, Germany). They performed one colonoscopy and one polypectomy in the new trainer and in the ELITE. After each model, a questionnaire was answered assessing suitability of the model for different training levels, realism of simulation and subjective stress during procedure. For the polypectomy, cube-shaped foam sponges with a side length of approx. 0.5 cm were positioned in a distance of $\sim$ 3 cm from the caecum of the CoVaMo and in the ELITE. The participants had to find these foam sponges during endoscope retraction, grasp and retrieve them, using an endoscopic loop. One assistant supported all the test persons in manipulating the loop for the polypectomy. For each participant, we recorded the suction pressure achieved per side (colon ascendens and colon descendens), the times required to maneuver from one anatomical landmark to another (i.e., from anus to flexura sinistra, to flexura dextra, into the caecum) and to grasp the sponge with the loop and retract the endoscope from the colon (polypectomy) (Fig. 6).

The adhesion force between the tissue and the inlay created by the vacuum was then calculated for both sides individually by:

$$ F^{D/A}_{{{\text{total}}}} = \mathop \sum \limits_{i = 1}^{5} F_{i}^{{{\text{chamber}}}} = \mathop \sum \limits_{i = 1}^{5} \Delta p* A_{i} $$

($\Delta p$: Difference between atmospheric and system pressure; *A*: suction area per chamber; *i:* number of chambers).

Table 1 lists the participants’ specialization, number of performed colonoscopies and polypectomies, and the years of employment

**Table 1:** List of all test persons indicating participants’ ID, specialization, number of colonoscopies and polypectomies performed, years of experience and experience level. The participants are ordered by their experience level (Abbreviations: assistant doctor: Ass. Doc; Surgeon: specialization in visceral surgery)

Test person ID	Profession	# Colonoscopies	# Polypectomies	Years of experience	Experience level
2	Student	0	0	0	Novice
5	Ass. Doc	0	0	3	Novice
8	Ass. Doc	0	0	1	Novice
9	Surgeon	0	0	14	Novice
11	Ass. Doc	10–20	0	2	Novice
3	Surgeon	50	5	10	Novice
1	Surgeon	400	150	8	Advanced
7	Surgeon	700	250	20	Advanced
4	Endoscopist	4000–5000	400–500	20	Expert
6	Surgeon	2000–5000	< 100	40	Expert
10	Endoscopist	8000	1500	15	Expert

### Questionnaire

The questionnaire comprised 19 rating questions using a Likert Scale, including 5 response options each, ranging from 1) hardly realistic, to 5) very realistic. The questions were divided in 5 categories assessing realism of the visual impression (appearance of mucosa, endoscopic image, orientation within the lumen), the haptic impression (friction, resistance during endoscope advancement, elasticity, gliding behavior within the lumen, haptic overall feeling), depiction of colon shape (length, angulation, diameter of the colon, 3D-positioning, connection to abdominal cavity), endoscope/instrument handling (range of movement, endoscope control) and advancement forces required. In addition, we assessed the subjective estimation of suitability for different training levels and effort required (stress, fatigue, concentration) by the participants. For all subgroups, a maximum of five points (very realistic) could be reached, except for the dimension “Advancement force required”. Here, a maximum of three points was achievable, with one point corresponding to a significantly higher or lower force and two points for slightly higher or lower forces than in reality.

### Statistical analysis

Statistical analysis was performed on the times measured between anatomical landmarks, total procedure time and time to perform the polypectomy.

For the results of the time measurements, we performed a Shapiro Wilk test, to see, whether each of the data sets can be described by a Gaussian distribution. As some of the sets were not, and others were normally distributed, we performed both, a less restrictive non parametric test (Wilcoxon-signed rank) and a more restrictive parametric test (paired *t*-test) to analyze our data. At first, the ${\varvec{H}}_{0} - {\varvec{Hypothesis}},$ whether there is any difference between the times measured, was evaluated using a MANOVA test, which revealed a statistically significant difference between the groups (${\varvec{\alpha}}_{{{\text{MANOVA}}}} = 0.05$). For further specification, we performed another analysis (level of significance ${\varvec{\alpha}} = 0.05$), comparing all groups against each other. For each of the resulting five ${\varvec{H}}_{0} - {\varvec{Hypotheses}}$, we performed a Bonferroni-correction (*p*-value adjustment).

## Results

### Adhesion forces

For CA the mean adhesion force achieved was 37 $\pm 7 {\mathbf{N}}$, and for CD 30 $\pm 15$ N. The mean suction force per chamber at the CA was 8 $\pm 1 {\mathbf{N}}$ N and at the CD 6 $\pm 3 {\mathbf{N}}$.

### Questionnaire

**Fig. 7:**
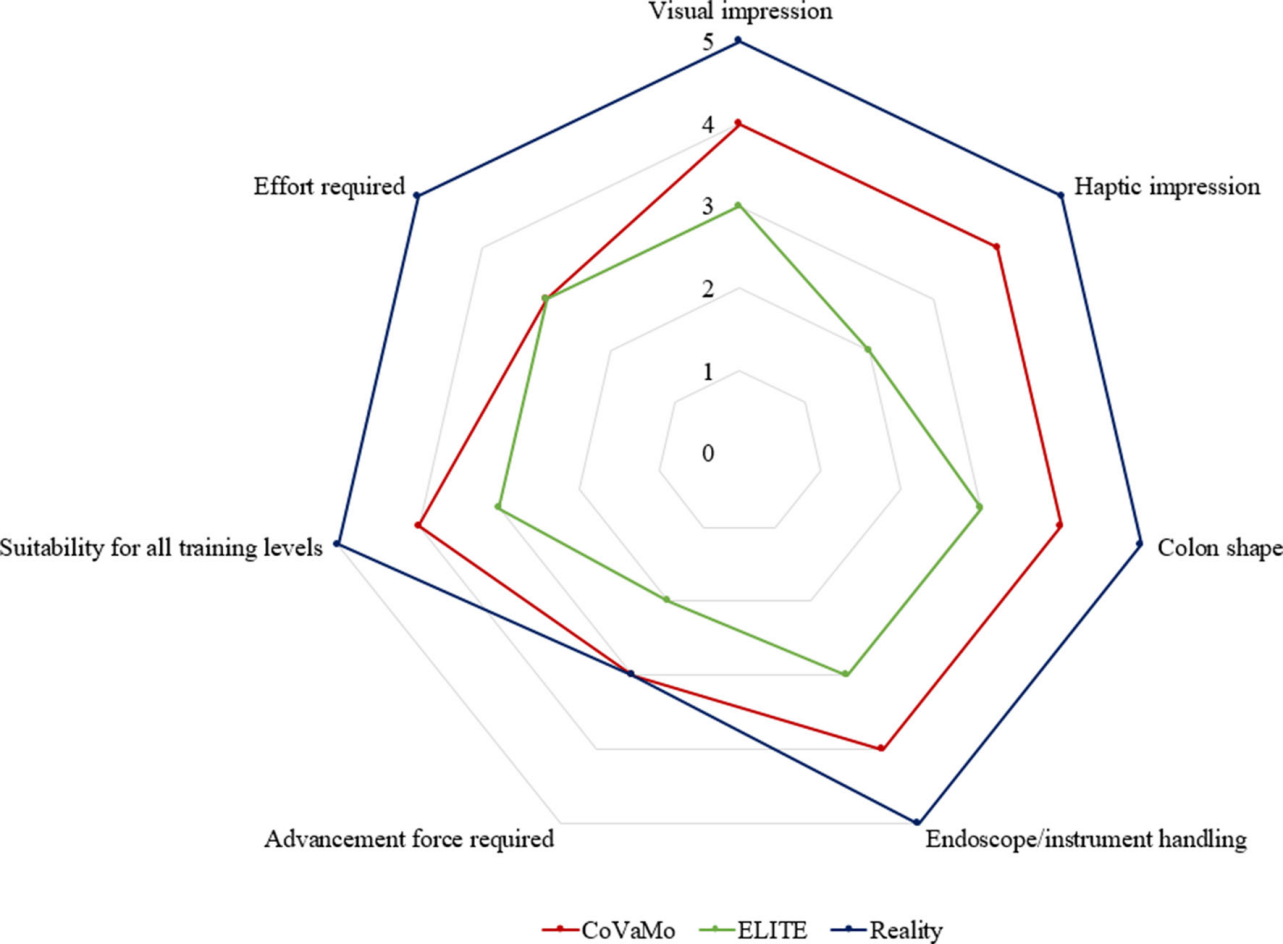
Spider plot visualizing the results of the questionnaire which was used to evaluate proximity to reality for the ELITE and the CoVaMo. Green: ELITE; Red: CoVaMo; Blue: Reality

The questionnaire evaluated the realistic character of CoVaMo in direct comparison with the validated ELITE phantom. We assessed the scores of each subgroup as illustrated in Fig. 7.

The blue graph reflects the maximum score to be achieved in all dimensions. The red graph represents the questionnaire scores for the CoVaMo and the green graph for the ELITE.

### Maneuver time measurement

**Fig. 8:**
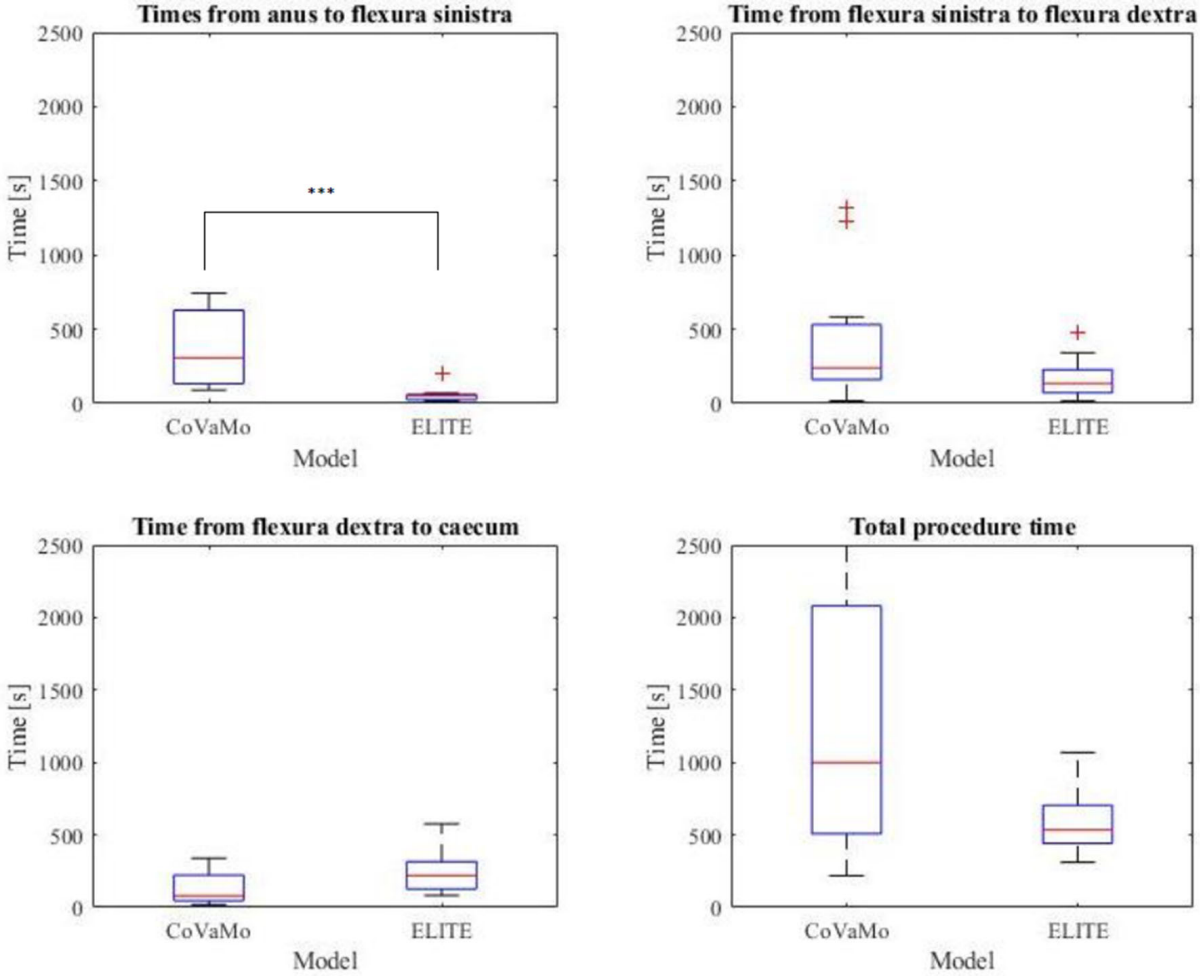
Duration required to maneuver from one landmark to another and for the entire procedure in CoVaMo and ELITE, for all participants (*n* = 11). The time required to pass from anus to flexura sinistra was statistically significantly longer in CoVaMo than in the ELITE. (Boxplot: central line: median, top and bottom boundaries: $$25th $$ and $$75th$$ percentiles of the measured data. Whiskers extend to the most extreme data points excluding outliers, marked by ‘ + ’. Outliers: values more than 1.5 times the interquartile range away from the top or bottom of the box. Three asterix indicate a statistically significant difference between CoVaMo and ELITE with respect to significance level 0.01)

**Fig. 9:**
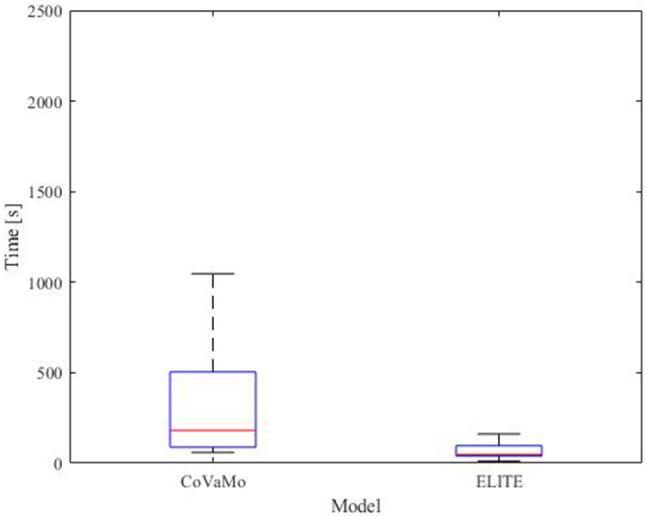
Time required by the participants (*n* = 11) for the polypectomy in CoVaMo and ELITE

We recorded the times required to advance the endoscope from the anus to flexura sinistra, from flexura sinistra along the transversum to flexura dextra, and from flexura dextra into the caecum. Furthermore, we evaluated the times for the polypectomy and for the entire procedure (colonoscopy including polypectomy) for both models for all participants (Table 2, Figs. 8 and 9).

**Table 2:** Times required by the participants to perform the tasks of the colonoscopy (*C*:CoVaMo; *E*:ELITE; *R*:Reality). The table gives mean values averaged over all participants, the corresponding standard deviations and median values

Time [s]	Flexura sinistra		Flexura dextra		Caecum		Total procedure time		Polypectomy	
	*C*	*E*	*C*	*E*	*C*	*E*	*C*	*E*	*C*	*E*
Mean	377	58	426	168	135	241	1271	599	333	67
Standard deviation	244	49	429	133	100	138	893	208	313	41
Median	307	53	239	135	79	219	999	535	181	48

The Wilcoxon-signed rank and the paired *t*-test both showed, that the time required to maneuver from the anus to flexura sinistra was statistically significantly longer for CoVaMo than for ELITE. There was no statistically significant difference for the other times measured, however the same tendency can also be observed for the segment between flexura sinistra and flexura dextra, the entire procedure time and the polypectomy (Figs. 8 and 9).

**Fig. 10:**
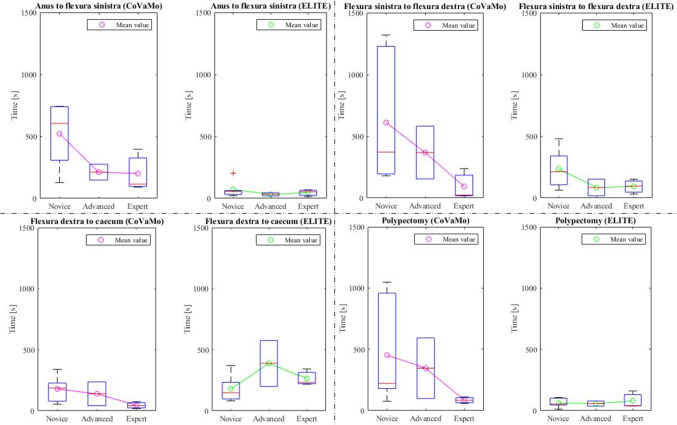
Times required to fulfill the tasks 1–4 (Fig. 6) in CoVaMo (Magenta) and ELITE (Green) (Boxplot: central line: median, top and bottom boundaries: 25th and 75th percentiles of the measured data. Whiskers extend to the most extreme data points excluding outliers, marked by ‘ + ’. Outliers: values more than 1.5 times the interquartile range away from thetop or bottom of the box). Groups are categorized according to the experience levels of the subjects

**Fig. 11:**
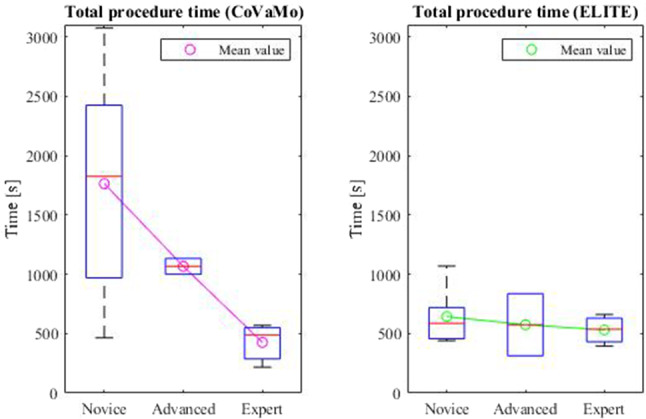
Dependence between the experience level and the time required to perform the entire procedure in the CoVaMo and ELITE, averaged over all test persons. The errorbars illustrate the standard deviation

Furthermore, we assessed the measured procedure time with respect to the participants’ experience levels (Figs. 10 and 11). Therefore, we plotted the times measured for the test persons in each group (boxplots), for CoVaMo (magenta) and ELITE (green) and indicated mean values per group by an “o”. For a better visualization of the trends observed, we connected the dots by lines.

## Discussion

The field of endoscopic trainers is diverse, comprising real animal models, computer-based simulators, purely mechanical trainers or hybrid solutions. However, there is a lack of trainers, which realistically simulate biomechanical restrictions by simultaneously providing a flexibly modifiable environment for the evaluation and training of new therapeutic, endoscopic procedures and instruments without injuring the colon specimen. Training variability with other ex vivo trainers is often limited due to inflexible [16], simplified and/or standardized setups, which do not offer the possibility to train with different anatomies. Furthermore, most systems simulate fixation of the intestine to the model only at the exit (anus) or rudimentarily by using meshes or shaping wires along colon descendens and colon ascendens [2, 4, 5]. This may serve for defining organ progression throughout the abdominal replica, but does not represent biomechanical restrictions. The Tübinger MIC Trainer and the KindHeart Colorectal Surgery Simulator (${\mathbf{KindHeart}}^{{{\mathbf{TM}}}}$, Chapel Hill, North California) are two of the very few simulators, realistically modelling adhesion of colon material to the dorsal wall [6, 16]. The latter one consists of a tissue cassette, delivered on ice and in a vacuum-sealed packaging. It comprises an organ package with contiguous structures and mesenteric vessels are used to simulate pulsation during laparoscopic colon resection. The simulator is sold and prepared by ${\mathbf{KindHeart}}^{{{\mathbf{TM}}}}$ and delivered to the customer at least 24 h before usage, as the system has to thaw. The trainer most closely simulates the biomechanical animal tissue properties, however, the training capacity per single system is limited due to the prepared tissue package configuration. For the Tübinger MIC Trainer, the adhesion of colon to the dorsal abdominal wall is simulated by sewing organs to a metallic mesh [6]. This allows for a higher customizability compared to the ${\mathbf{KindHeart}}^{{{\mathbf{TM}}}}$ trainer. However, as high forces occur during endoscopic interventions traumatic fixation weakens the tissue and may favor sample tearing. To enable multiple training sessions and a flexibly adjustable setup without weakening the specimen, our approach establishes a modifiable and measurable adhesion force, by creating a vacuum in chambers underneath the colon specimen. By measuring the pressure achieved during experiments, we examined a mean adhesion force of 37 $\pm 7 {\mathbf{N}}$ at the CA, and 30 $\pm 15$ N at the CD. For a comparison to real biomechanical restrictions in an organism, we determined the force required to tear pig colon off its mesentery in an animal experiment. With a sample size of *n* = 5, we determined a mean force of 15 $\pm 6 {\mathbf{N}}$. Therefore, the adhesion forces of our model were higher than the threshold assessed in the pig. By tuning the vacuum, using different pumps or by opening/closing of one or several chambers, the adhesion forces can be modified. In this way, different preconditions, such as partial/full or unilateral/bilateral mobilized bowels could be modeled. This is supposed to allow simulation of diverse interventions and to address different skill levels.

In addition, vacuum as a fixation mechanism makes the adhesion independent of lumen diameter and wall thickness variations and does not hamper endoscope or instrument movement within the lumen. The short preparation time per test person enables time-efficient use, also in large scale studies. Changing specimens in the CoVaMo takes about 5 min, while the most time consuming step is the preparation of the anus. Establishing adherence and adopting the anatomical conditions can be achieved within seconds, by pressing the tissue onto the suction surfaces and switching on the vacuum pumps. In contrast, the preparation of the Tübinger MIC Trainer takes about 20 min [17].

The analysis of the questionnaire results revealed that CoVaMo was considered more realistic than the ELITE, with respect to overall anatomical representation, including visual, haptic impression and colon shape, as well as endoscopic procedure simulation (Fig. 7). Furthermore, the participants rated CoVaMo more suitable for different training levels than ELITE. Practicing on our model required comparable effort as in the ELITE. Concerning the forces needed to advance the endoscope, the participants rated CoVaMo more realistic than the ELITE, as well. This is also indicated in Fig. 10. Except for the distance between flexura dextra and caecum, the subjects always needed more time to perform the tasks in the CoVaMo than in the ELITE. Due to the rubber-like colon material in the ELITE, the friction between organ and the fully inserted endoscope is quite high. Therefore, the subjects had difficulties to advance the colonsocope into the caecum. Six out of eleven test persons were not able to push the colonoscope all the way forward into the end of the caecum. The remaining distance was $\sim$ 2–3 cm. For the CoVaMo, all test persons were able to reach the caecum.

We furthermore assessed the correlation between the subjects’ level of experience and the time needed to perform a task (Figs. 10 and 11). The assumption was that for less trained participants, it is harder and takes more time to navigate the endoscope to the caecum and to perform the polypectomy. We observed a decrease in mean time with increasing experience level in the CoVaMo for all tasks (Figs. 10 and 11). For the ELITE, in contrary, there is hardly any variation detectable between the experience levels. Additionally, statistical analysis showed, that participants needed significantly more time to pass from the anus to flexura sinistra in the CoVaMo than in the ELITE. This segment is the most challenging during colonoscopy, due to the high flexibility and range of motion at the sigma. This leads us to the conclusion, that the CoVaMo allows for a more realistic distinction between the different skill levels.

In both figures (Figs. 8 and 9), we also observed larger standard deviations in all groups and for all maneuvering tasks for the CoVaMo than for the ELITE. As in CoVaMo participants are required to operate all endoscope features, such as insufflation to maintain a stable lumen, aspiration for lumen visibility and endoscopic tip position control, encountering all typical environment and procedure-related complications during navigation throughout the colon, it is more demanding and subjects are challenged by multiple tasks in parallel. Therefore, we have a larger scattering in all groups, in particular for the least trained category of novices (Figs. 8, 9, 10, and 11). We assumed, that variations in numbers of colonoscopies performed have a higher impact on the performance in this group, than in the others, due to the overall lack of experience and practice. The statistically proven prolonged duration to complete certain tasks may also reflect the multimodal challenges of CoVaMo, realistically simulating real procedures. To draw the comparison to reality, the total procedure times of 20 standard colonoscopies (without polyp resection) performed by experts (different from our test persons) at our hospital were evaluated. The times required to perform the entire colonoscopy were very similar for CoVaMo (1271 s ± 893 s) and reality (1435 s ± 545 s). In contrast, the mean total procedure time in the ELITE was remarkably lower (599 s ± 280 s).

## Conclusions

We were able to prove resemblance to reality of our trainer in all assessed dimensions (adhesion force measurement, questionnaire, time measurements). By simulating mechanical restrictions for the retroperitoneally located intestinal segments using vacuum, it is possible to establish measurable and modifiable adhesion forces. With manufacturing costs of approximately 260 Euros for the model (including the distributor valves for connection to the vacuum pumps, excluding vacuum pumps), the workflow can be easily adapted to any arbitrary mechanical model or ex vivo simulator, to cost-efficiently top up the trainer and enable a more diverse training setup.

## Footnotes

1 DeLegge Medical, Inc.: TRAY PURCHASE–EndoExpert Tray, https://www.organsbydesign.com/products/endoexpert-tray-replaces-endoeddie, last access date: 23.09.2022.

2 EndoSim, LLC, 41 Main St., Bolton, MA 01,740, USA; EASIE-R1 AND R3, ColoEASIE-2 simulators; https://endosim.com/gastroenterology; last access date: 23.09.2022.

3 Formlabs; https://support.formlabs.com/s/article/Using-Durable-Resin?language=en_US; last accessed: 21.09.2022.
